# Metal Pneumonitis from “Non-toxic” Decorative Cake Dust Aspiration: A Case Report

**DOI:** 10.5811/cpcem.7220

**Published:** 2024-07-28

**Authors:** Taylor Sanders, Mitchell Hymowitz, Christine Murphy

**Affiliations:** * Atrium Health’s Carolinas Medical Center, Department of Emergency Medicine, Division of Medical Toxicology, Charlotte, North Carolina; † Louisiana State University Health Sciences Center, School of Medicine, Emergency Medicine Residency Program, Baton Rouge Campus, Baton Rouge, Louisiana

**Keywords:** case report, cake dust, bronze, metal pneumonitis, pediatric

## Abstract

**Introduction:**

Metallic luster dusts are decorative agents for cakes and other confections. While some powders are labeled “non-edible,” they are also marketed as “non-toxic.” We present a case of a child who developed acute metal pneumonitis after accidental aspiration of metallic luster dust.

**Case Report:**

A four-year-old presented to the emergency department (ED) in respiratory distress after attempting to ingest gold decorative metallic luster dust. In the ED she was placed on supplemental oxygen. Her initial chest radiograph (CXR) was unremarkable. Her condition worsened despite high-flow nasal cannula oxygen, and she was intubated. A repeat CXR revealed patchy perihilar and peribronchial opacities. While receiving aggressive ventilatory support, her CXR worsened over the next 48 hours as bilateral interstitial and alveolar opacities progressed, likely representing acute metal pneumonitis with acute respiratory distress syndrome (ARDS). She remained intubated until hospital day (HD) 5, requiring supplemental oxygen until HD 9. She was discharged home on HD 10. A CXR obtained four months later demonstrated increased interstitial markings throughout both lungs with overinflation and subsegmental atelectasis. The patient had persistent dyspnea upon exertion, with pulmonology documenting that her symptoms were likely sequelae from inhalation of the cake luster dust.

**Conclusion:**

Non-edible metallic cake dusts are toxic. “Non-edible” labeling does not convey the health risks associated with handling by children, as evidenced by this case of metal pneumonitis with associated ARDS and chronic pulmonary disease. Accordingly, this descriptor should be abandoned for these products, and physicians should be aware of this potential complication.

## INTRODUCTION

Luster dusts are increasingly popular decorative agents for cakes and other confections. Like decorative glitters, some luster dusts are safe for use on food and contain ingredients such as sugar, cornstarch, maltodextrin, and color additives specifically approved for use as food by the United States Food and Drug Administration (FDA).^1^ These products will typically display the descriptor “edible” on package labeling and must include a list of ingredients per the Federal Food, Drug, and Cosmetic Act and the Fair Packaging and Labeling Act.^2^ Other luster dusts are simply shavings of one or more metals, including aluminum, barium, chromium, copper, iron, lead, manganese, nickel, and zinc.^3^ These metallic luster dusts are not intended for human consumption but are sold online, in grocery stores, and in bakeries, often adjacent to the edible varieties. Due to their intended use as decorations only, these metallic cake dusts do not meet the FDA definition for food additives and are not subject to its regulations.^4^ While most manufacturers will include “non-edible” labeling, they are also marketed as “non-toxic” and can be quite similar in appearance to the edible decorative agents. We present a case of a child who developed acute metal pneumonitis after accidental aspiration of metallic luster dust.

## CASE REPORT

**Image 1:**
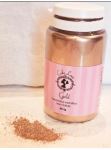
Gold decorative metallics luster dust bottle and contents.

A four-year-old female with no past medical history presented to the emergency department (ED) shortly after ingesting gold decorative metallic luster dust (Image 1). The patient’s mother reported she was in a nearby room when she heard the patient suddenly begin coughing and choking. When she came to investigate, the patient was holding the bottle with evidence of the cake dust both surrounding and within her nose and mouth, prompting the mother to immediately bring her to the ED.

On arrival, the patient was tachypneic (respiratory rate of 27 breaths per minute) and coughing, and appeared to be in distress. She had coarse breath sounds and a room air oxygen saturation of 88% (reference range 95–100%). She was placed on heated high-flow nasal cannula delivering 40% oxygen at 16 liters per minute (L/min), which initially increased her oxygen saturation to 99%. A chest radiograph (CXR) was obtained and interpreted as unremarkable. An initial venous blood gas (VBG) revealed a partial pressure of carbon dioxide (pCO2) of 29 millimeters of mercury (mm Hg) (35–45 mm Hg) with pH 7.39 (7.35–7.45). While in the ED, her respiratory rate increased to 36 breaths per minute, her mental status declined, and she developed diffuse crackles on chest auscultation. A repeat VBG 65 minutes after arrival (45 minutes after initial VBG) revealed pCO2 of 58 mm Hg with pH of 7.22.

**Image 2:**
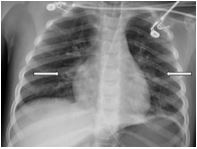
Chest radiograph obtained 148 minutes after arrival revealing bilateral patchy perihilar and peribronchial opacities (arrows).

With obtundation and a worsening respiratory acidosis, she was intubated and placed on synchronized intermittent mandatory ventilation with a fraction of inspired oxygen (FiO2) of 75% and positive end-expiratory pressure of 7 mm Hg. A post-intubation CXR, performed approximately 148 minutes after arrival (98 minutes after the initial CXR), revealed bilateral patchy perihilar and peribronchial opacities (Image 2). There were no acute complications with the intubation, and the patient remained hemodynamically stable while being transferred to the intensive care unit (ICU).

**CPC-EM Capsule**

What do we already know about this clinical entity?

*Metal pneumonitis is the most severe acute complication of metal inhalation, and its occurrence is not confined to the workplace or one specific metal.*

What makes this presentation of disease reportable?

*The case highlights the risk of severe lung injury from inhalation of a household product, for which no warning to consumers is disclosed by the manufacturer or the US Food and Drug Administration (FDA).*

What is the major learning point?

*Not all food items are regulated by the FDA or safe for handling by children. Severe inhalational injury can occur from metallic cake-dust products.*

How might this improve emergency medicine practice?

*Knowledge of lesser-known household agents and their potential to produce significant injury is important for emergency physicians.*

**Image 3:**
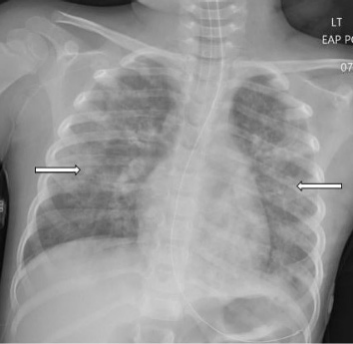
Chest radiograph on hospital day three showing worsening bilateral infiltrates and progression of mixed interstitial and alveolar opacities (arrows).

In the ICU she received continued mechanical respiratory support and intermittent diuresis with furosemide to maintain neutral fluid balance. The infiltrates seen on CXR worsened over the next three days with progression of mixed interstitial and alveolar opacities (Image 3). Despite a worsening CXR appearance, the patient maintained adequate oxygenation with ventilator settings of FiO_2_ of 30% and positive end-expiratory pressure of 5 mm Hg. She had intermittent fevers and leukocytosis, with a maximum white blood cell count of 26 x 10^3^ cells per microliter (μL) (reference range 4.5 x 10^3^ – 11 x 10^3^ cells/μL); however, antibiotics were deferred. There was no evidence of extrapulmonary organ damage on clinical exam or laboratory testing throughout the duration of her hospitalization. Two respiratory pathogen panels testing for 22 pathogens obtained on hospital days (HD) 2 and 4 were negative, as were blood, urine, and sputum cultures. She was extubated on HD 5. Post-extubation, she required 35–40% FiO_2_ via heated high-flow nasal cannula at 8–10 L/min for an additional two days as her CXR improved. She required supplemental oxygen until HD 9 and was discharged home the following day.

**Image 4:**
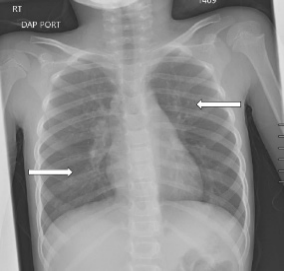
Chest radiograph at five months after discharge demonstrating increased perihilar and peribronchial markings bilaterally and mild reticular nodular opacity involving both lungs (arrows).

The patient was referred to both cardiology and pulmonology for outpatient evaluation by her primary care physician four months after her hospitalization. Her chief complaints at these follow-up visits included decreased oxygen saturation readings at home, dyspnea on exertion, decreased appetite, and fatigue. Cardiology performed a cardiac ultrasound, which was normal, and did not believe her symptoms were cardiac in nature. A CXR obtained five months later demonstrated increased perihilar and peribronchial markings bilaterally and mild reticular nodular opacity involving both lungs (Image 4). She was diagnosed with moderate persistent reactive airway disease which, per her treating pulmonologist, was likely a sequela of her acute lung injury from inhalation of the cake luster dust. At greater than one year since hospitalization, she continued to be prescribed budesonide/formoterol twice daily and rescue albuterol as needed.

## DISCUSSION

The cake luster dust in this case consists of a mixture of gold-appearing flakes and dust with a fine texture (Image 1). Online vendors note this product is “non-toxic”; “bronze based powders” are listed under ingredients. Bronze alloys, as a component of the cake dust, commonly consist of roughly 88% copper, 12% tin, and a trace amount of other metallic components. While this patient did not develop signs of heavy metal toxicity, there are at least seven reported cases of acute metal toxicity following ingestion of decorative cake dust.^3^ As in this case, there have been other published reports of inhalational exposures to metallic cake dusts. A recent case series describes three inhalational exposures leading to metal fume fever-like syndromes.^5^ In all three cases, symptoms improved with bronchodilators and resolved 48 hours after exposure. A 2022 case report of metal fume fever from cake dust described a preschool-aged male with acute inhalational lung injury who required high-flow nasal cannula (18 L/min, 43% oxygen) for 24 hours. He also had a persistent oxygen requirement for nine days after exposure.^6^

Metal fume fever is typically associated with inhalation of metal oxides like zinc and aluminum oxides that are formed when metals are heated through processes such as welding. Metal fume fever is one of several terms describing what is classically a “subclinical alveolitis not apparent on chest radiograph or pulmonary function tests except in unusually severe cases.”^7^ It is “associated with a neutrophilic predominance on bronchoalveolar lavage and a systemic leukocytosis” with short-lived clinical effects, similar to a flu-like illness, and is unlikely to lead to sequelae.^7^ Alternatively, exposures resulting in a prolonged course (greater than 72 hours) with pulmonary infiltrates on CXR and respiratory failure more likely represent acute metal pneumonitis with or without acute respiratory distress syndrome (ARDS).

High concentrations of several inhaled metallic dusts containing copper and mercury have been shown to cause acute pulmonary damage, resulting clinically in chemical pneumonitis and, in some cases, ARDS.^8^–^9^ Case reports have also described chronic pulmonary manifestations including pulmonary fibrosis and hyperreactive airway disease.^10^ Our case involves a notably severe clinical course with significant radiologic evidence of acute lung inflammation and both clinical and radiographic evidence of persistent pulmonary abnormalities, which we believe is consistent with metal pneumonitis.

## CONCLUSION

This case highlights that non-edible metallic cake dusts are easily perceived as lacking toxicity. These products are sometimes difficult to distinguish from edible cake dusts made from sugar and are often sold alongside them in retail settings. While “non-edible” labeling is frequently used, these same containers also display the words “non-toxic.” This does not adequately convey the health risks associated with improper handling or provide purchasers with a warning regarding which portions of a confection may be dangerous to eat. The inadequacy of this labeling is evidenced by this case of metal pneumonitis with associated ARDS and chronic pulmonary disease. Accordingly, “non-toxic” should be abandoned as a descriptor of these products, and both consumers and treating physicians made aware of potential complications from inadvertent exposures.
